# Innovaciones sociales para mejorar la salud

**DOI:** 10.7705/biomedica.6725

**Published:** 2022-09-02

**Authors:** Luis Gabriel Cuervo-Amore, Magaly M. Blas

**Affiliations:** 1 Asesor principal en Investigación para la Salud, Organización Panamericana de la Salud (OPS/ OMS), Washington D.C., , USA; doctorando en Metodología de la Investigación y Salud Pública, Universitat Autònoma de Barcelona, Barcelona, España Organización Panamericana de la Salud (OPS/ OMS) Washington D.C. USA; 2 Profesora asociada, Facultad de Salud Pública y Administración, Universidad Peruana Cayetano Heredia, Lima, Perú; directora, Mamás del Río Universidad Peruana Cayetano Heredia Facultad de Salud Pública y Administración Universidad Peruana Cayetano Heredia Lima Peru

Las innovaciones sociales en salud son procesos en los que las comunidades y los innovadores, en forma colaborativa, crean e implementan soluciones prácticas a los problemas de la salud y sus factores determinantes. Usualmente, influyen sobre los sistemas y servicios de salud locales ^1^^,^^2^.

Existen múltiples definiciones de *innovación social*, las cuales concuerdan en que se producen cambios resultantes de la creación colectiva de conocimientos desarrollados con la comunidad, o por ella, cambios que tienen un enfoque holístico, deben ser sostenibles y dejar capacidades instaladas; el liderazgo suele ser compartido entre la comunidad y otros actores ^2^.

En la práctica, las innovaciones representan la traducción integrada del conocimiento en la que, de principio a fin, se aprende, se ajusta y se mejora, siguiendo un diseño centrado en las personas. Con frecuencia, estas innovaciones se insertan en los planes, programas y políticas públicas ^2^^,^^3^. Estos conceptos los ilustraremos con ejemplos.

Para conocer las innovaciones, se requiere mirar procesos y resultados hilando las historias que las definen; son ricas en contexto y complejidad en su evolución ^2^^,^^4^. La *Social Innovation in Health Initiative* (SIHI) y el *Café Virtual* del Centro Internacional de Entrenamiento e Investigaciones Médicas (CIDEIM), coleccionan historias así.^1^ ^2^

En Colombia, las familias afectadas por el Zika -apoyadas por investigadoras del Instituto Nacional de Salud- aportaron evidencia para mejorar la calidad de los servicios de salud. Las evidencias sustentaron nuevas políticas públicas acordes con las necesidades de esas familias y niños afectados por el virus del Zika ^5^.

*Mamás del Río* es una innovación peruana que ilustra bien los elementos de la innovación social y su apuntalamiento en la investigación, con una historia y un contexto documentado que llega a diferentes audiencias ^6^^-^^9^.

En el 2015, investigadores de la Universidad Peruana Cayetano Heredia visitaron el río Marañón en la Amazonía peruana, una de las regiones con los peores indicadores de salud materna y neonatal ^10^. Junto con la Dirección Regional de Salud (DIRESA) de Loreto, visitaron 13 comunidades en el distrito de Parinari. Se ganaron la confianza de las comunidades y aprendieron de la gran prevalencia de partos domiciliarios atendidos sin personal entrenado, y del pobre acceso a la atención de salud de calidad para los controles prenatales, partos y emergencias. Además, notaron que ni la morbilidad ni la mortalidad se reflejaban adecuadamente en los registros oficiales; como si las personas no existieran ^8^^,^^10^.

Investigaron las razones con la comunidad y DIRESA, y debatieron la relevancia y las opciones para atender estas situaciones en forma sostenible y adecuada al contexto, de forma que generaran capacidades locales instaladas y perdurables que, de resultar efectivas, se insertaran en los planes, programas y políticas públicas ^11^^,^^12^.

Concluyeron que la comunidad necesitaba contar con capacidad instalada local para responder a sus problemas de salud. Entonces, *Mamás del Río* entrenó a agentes comunitarios de salud, habilitándolos para vigilar la gestación, hacer visitas domiciliarias a mujeres gestantes y recién nacidos, y articular a las comunidades con los servicios de salud. Con el apoyo de la Universidad, autoridades locales y DIRESA, se promovió el entrenamiento de parteras y de personal de salud, y la sensibilización de la comunidad. Todo esto contaba con un sistema de supervisión continua ^6^^,^^7^^,^^9^.

La estrategia *Mamás del Río* y la municipalidad de Parinari, en conjunto, promovieron el registro de los pobladores y de los recién nacidos. La ciudadanía recibió certificados de nacimiento y documento nacional de identidad. Esto los habilitó para reclamar sus derechos ciudadanos y aparecer en las estadísticas, e ilustra el enfoque holístico con el que se enfrentan los factores determinantes de la salud ^9^.

Las comunidades rivereñas carecen de servicios de agua y electricidad, pero tienen telefonía celular con datos. Los agentes comunitarios de salud capacitados recibieron teléfonos celulares con servicio de datos, que les permitían estar en contacto con los establecimientos de salud. Cada comunidad instaló un panel solar y un receptáculo artesanal donde permanecía el teléfono asignado al agente. Eventualmente, se remplazaron por tabletas digitales que operan fuera de línea y ofrecen más funciones: guían las visitas de los agentes, y ofrecen imágenes, vídeos y contenidos estandarizados creados con las comunidades. También, recaban información sobre decisiones en el sector salud ^8^. Esto ejemplifica el involucramiento y el compromiso de las comunidades en el desarrollo y en la implementación de las innovaciones.

Las reuniones de los agentes comunitarios de salud con los trabajadores locales de salud generaron un sentido de equipo y comunidad, y las consecuentes confianza y comunicación. El programa ganó reconocimiento e integración con los servicios de salud.

Hoy, las comunidades abogan para que se reconozca a estos agentes comunitarios como trabajadores del sistema de salud, buscando un cambio en la política para consolidar la integración al sistema de salud y hacer sostenible la estrategia de las *Mamás del Río* (comunicación personal, Magaly Blas, 2022).

El interés por las *Mamás del Río* se propagó a más comunidades, entidades filantrópicas, y financiadores nacionales y extranjeros. Esto apoyó su escalamiento a 84 comunidades en tres distritos de Loreto, extendiéndose a lo largo de 350 kilómetros de ríos ^8^. El empoderamiento de las comunidades y el interés pueden haber contribuido, además, al avance de programas y políticas públicas ^11^. Por ejemplo, las cancillerías de Colombia y Perú, viendo los resultados, se interesaron en escalar el programa a la frontera binacional. Esto ilustra la integración con las políticas, planes y programas, consolidando el cambio y generando sostenibilidad ^7^^,^^9^^,^^12^.

*Mamás de la Frontera* es la extensión de *Mamás del Río* a la frontera peruano-colombiana, respaldada por las cancillerías y los ministerios de Salud binacionales, y las agencias multilaterales e intergubernamentales. *Mamás de la Frontera* cubre 30 comunidades fronterizas del río Putumayo en un trayecto fluvial de 400 kilómetros ^9^^,^^13^. Los desarrollos infieren la transición y ampliación de roles que tienen los innovadores a lo largo del proceso ^12^.

Los innovadores suelen demostrar adaptabilidad y esto se evidenció durante la pandemia por COVID-19. Varias de las innovaciones sociales reconocidas por la SIHI a nivel mundial y en las Américas, fueron críticas en la reacción ante la pandemia por COVID-19 ^14^. Cuando la pandemia amenazó la Amazonía, los agentes comunitarios de salud de *Mamás del Río* y *Mamás de la Frontera* se capacitaron para aportar información de calidad a sus comunidades, apoyar la prevención, facilitar la remisión y seguimiento de pacientes, y fomentar la vacunación contra la COVID-19 ^7^^,^^14^^,^^15^. Esto ilustra la forma como un ecosistema favorable puede potenciar las oportunidades de innovación ^12^^,^^14^.

En 2021, la red americana de SIHI, la SIHI-LAC (*Social Innovation in Health Initiative in Latin America and the Caribbean*), con apoyo de la Organización Panamericana de la Salud (OPS), hizo un llamado público para identificar innovaciones sociales que estuvieran contribuyendo a mejorar la salud pública y la prestación equitativa de servicios de salud, durante la pandemia^3^. Se presentaron 106 aplicaciones elegibles, cuatro recibieron mención honorífica y cuatro fueron reconocidas como ganadoras ^16^. Estas innovaciones incluyen nuevas soluciones para mejorar la resiliencia de los sistemas de salud, e ilustran la riqueza de las acciones intersectoriales, los avances prácticos, el uso de tecnologías y los logros. La colección de recursos de la SIHI aporta detalles de los efectos sistémicos de docenas de innovaciones.

Hay muchas iniciativas que, como la SIHI, se enfocan en promover la innovación social para la salud mediante llamados (*crowdsourcing calls*), estudios de casos y apoyo al ecosistema de la innovación social ^1^^,^^17^^,^^18^.

La SIHI se inició en 2014, liderada por el TDR (*Special Program for Research and Training in Tropical Diseases*), con apoyo de destacadas organizaciones filantrópicas, académicas e intergubernamentales de diferentes continentes. ^4^

El TDR es cofinanciado por la OMS, la UNICEF, el Banco Mundial y el Programa para el Desarrollo de las Naciones Unidas (PNUD), y canaliza los fondos de la *Swedish International Development Agency* para apoyar la SIHI^5^.

El secretariado de la SIHI-LAC lo conforman el CIDEIM y la OPS, trabajando en colaboración con entidades académicas como la Universidad Nacional Autónoma de Honduras, la Universidad de Antioquia y la Universidad Icesi^5^.

La innovación social debe implementar soluciones. En la SIHI se buscan enfoques prácticos, intersectoriales y multidisciplinarios, que atiendan los factores determinantes sociales de la salud, y que promuevan el emprendimiento y el desarrollo social ^19^.

El impulso a la innovación social ha favorecido que se adopten estándares para los informes de las iniciativas y de la investigación que las respalda ^20^. También, ha facilitado intercambios entre innovadores y capacitadores para apoyar a los primeros al asumir nuevos roles, a medida que los proyectos evolucionan. Con frecuencia, quienes innovan van adoptando nuevos roles y deben ir desarrollando nuevas habilidades: cuidadores, capacitadores, coordinadores, embajadores, emprendedores, gerentes, movilizadores de recursos, negociadores, investigadores, mediadores, comunicadores sociales y educadores ^9^.

Por ello, los innovadores valoran especialmente el desarrollo de habilidades competencias personales (*soft skills*) que les faciliten la gestión y la sostenibilidad. Para ello, también participan en intercambios, encuentros con entidades filantrópicas y debates metodológicos sobre innovación social ^12^^,^^18^^,^^20^^-^^23^.

Un elemento central en la SIHI ha sido promover la divulgación científica y el intercambio de ideas. Las iniciativas reconocidas en los llamados son documentadas y se siguen generando lecciones sobre formas de facilitar el ecosistema de la innovación social ^12^.

Siendo SIHI una red mundial, el intercambio también promueve la adaptación y la adopción de las iniciativas en nuevos ámbitos, y se incentivan actividades de desarrollo para actores clave de las iniciativas ^12^. Por ejemplo, SIHI-LAC ha organizado talleres priorizados por los innovadores y SIHI ha organizado intercambios entre sus nodos regionales y con entidades filantrópicas, y con apoyo de la SESH Global (*Social Entrepreneurship to Spur Health*), la OPS, el TDR, la *Swedish International Development Agency* (SIDA) y el *British Medical Journal* (BMJ), produjeron en 2022 un suplemento sobre innovación social en salud en *BMJ Innovations*^6^.

Colombia ha figurado en estos avances, incluyendo los de la SIHI-LAC ^2^^,^^5^^,^^11^^,^^16^^,^^24^^).^ El CIDEIM y la OPS lideran el secretariado de la SIHI-LAC y apoyan el secretariado mundial que ahora está en Manila, Filipinas. La innovación social para la salud tiene larga tradición y respaldo en Colombia ^2^^,^^25^^-^^31^. En la SIHI, el liderazgo del CIDEIM y el respaldo de la Universidad de Antioquia y otras instituciones, le ha dado visibilidad internacional ^12^^,^^25^ ^5^.

La comunidad científica ha apoyado la innovación social para la salud, por ejemplo, desde la Misión Internacional de Sabios de Colombia, grupo de expertos nacionales e internacionales convocado por el gobierno para aportar a la política pública de educación, ciencia, tecnología e innovación ^31^. Las innovaciones sociales conllevan aprendizaje y pueden tener efectos sistémicos transformadores perdurables que se manifiestan en plazos largos.

Dicho grupo propone aprovechar la innovación social para fortalecer los sistemas y servicios de salud, vinculando la innovación, la ciencia y las comunidades. Incluyen la innovación social en la hoja de ruta e indicadores necesarios para cumplir los Objetivos de Desarrollo Sustentable (ODS) y lograr la sostenibilidad social y ambiental ^31^.

La innovación social es destacada por la Misión de sabios por transformar “de abajo hacia arriba, o en conjunto con actores del sistema actual” ^31^. También, consideramos que tiene otros efectos a largo plazo. En muchas de las comunidades en que se dan estas innovaciones, han sido escasas las oportunidades que tiene la comunidad y, en particular, la infancia y la juventud, de exponerse a innovadores e investigadores. Esos modelos y roles suelen serles nuevos y, por tanto, representan una oportunidad para inspirar las siguientes generaciones de innovadores y personas de ciencia, para que esté bien reflejada la riqueza y la diversidad de las sociedades.

Esta puede ser la situación para las poblaciones guatemaltecas en las que se ha interrumpido la transmisión de la enfermedad de Chagas al mejorar las viviendas con materiales y tecnologías autóctonas, con apoyo de la Universidad de San Carlos y liderado por la doctora Carlota Monroy ^32^, o para los habitantes de Sumapaz en la zona rural de Bogotá, donde científicos de la Universidad Nacional y de la SubRed Sur de la Secretaría de Salud apoyan a las innovadoras de la comunidad, mejorando la calidad y seguridad alimentaria, y el entorno ecológico ^11^^,^^24^.

El contar con más innovadores e investigadores provenientes de estas comunidades, quizás facilite atender agendas inconclusas, proteger logros y abordar nuevos retos. Bienvenidas las innovaciones y las personas que serán modelos para seguir entre las generaciones venideras. 

Nuestros agradecimientos a la doctora Mónica Padilla, asesora de Servicios y Sistemas de Salud de OPS, por la revisión del manuscrito.

## Footnotes

1 Social Innovation in Health Initiative. Social Innovation Cases. 2015-2022 https://socialinnovationinhealth.org/innovation-overview/

2 Corporación CIDEIM. Café Virtual (Playlist), Conducido por la doctora Diana María Castro Arroyave. 2018- 2022. https://tinyurl.com/CafeVirtual-InnovacionSocial

3 SIHI-LAC. Búsqueda de soluciones que durante la pandemia COVID-19 mejoren la salud pública y la entrega de servicios de salud con equidad. https://socialinnovationinhealth.org/identification/identificando-innovaciones-en-america-latina-y-el-caribe/

4 Alianzas de SIHI: https://socialinnovationinhealth.org/partner-overview/

5 Centros Regionales de Entrenamiento de TDR: https://tdr.who.int/home/our-work/strengthening-research-capacity/regional-training-centres

6 BMJ Innovations Special supplement on social innovations in health [Suplemento especial sobre innovaciones sociales en salud]. 16 de junio de 2022 https://www.bmj.com/company/social-innovations-for-health

## Lecturas recomendadas

- Abookire S, Plover C, Frasso R, Ku B. Health design thinking: An innovative approach in public health to defining problems and finding solutions. Front Public Health. 2020;8. Fecha de consulta: 18 de julio de 2022. Disponible en: https://www.frontiersin.org/articles/10.3389/fpubh.2020.00459
- Brown T, Wyatt J. Design thinking for social innovation. Dev Outreach. 2012. Fecha de consulta: 2 de abril de 2021. Disponible en: https://elibrary.worldbank.org/doi/abs/10.1596/1020-797X_12_1_29
- Domanski D, Howaldt J, Kaletka C. A comprehensive concept of social innovation and its implications for the local context - on the growing importance of social innovation ecosystems and infrastructures. Eur Plan Stud. 2020;28:454-74. https://doi.org/10.1080/09654313.2019.1639397
- Howaldt J, Kaletka C, Schröder A. Social entrepreneurs: Important actors within an ecosystem of social innovation. Eur Public Soc Innov Rev. 2016;1(2). Fecha de consulta: 31 de agosto de 2021. Disponible en: https://pub.sinnergiak.org/esir/article/view/43
- Howaldt J, Kopp R. Shaping social innovation by social research. En: Franz HW, Hochgerner J, Howaldt J, editors. Challenge social innovation: Potentials for business, social entrepreneurship, welfare and civil society. Berlin, Heidelberg: Springer; 2012. p. 43-55. Fecha de consulta: 3 de septiembre de 2022. Disponible en: https://doi.org/10.1007/978-3-642-32879-4_3
- Jull J, Giles A, Graham ID. Community-based participatory research and integrated knowledge translation: Advancing the co-creation of knowledge. Implement Sci. 2017;12:150. Fecha de consulta: 22 de marzo de 2021. Disponible en: https://doi.org/10.1186/s13012-017-0696-3
- Nicholls A, Simon J, Gabriel M, editors. New frontiers in social innovation research. London: Palgrave Macmillan UK; 2015. Fecha de consulta: 3 de septiembre de 2022. Disponible en: http://link.springer.com/10.1057/9781137506801
